# Supplementary material for: Discovering cognitive strategies with tiny recurrent neural networks
Source: Nature. 2025 Jul 2;644(8078):993–1001. doi: 10.1038/s41586-025-09142-4 (PMC12390849; doi:10.1038/s41586-025-09142-4)
Supplement: Supplementary file 2 — Reporting Summary [file 41586_2025_9142_MOESM2_ESM.pdf]

nature portfolio

Corresponding author(s): Marcelo G. Mattar

Last updated by author(s): Apr 22, 2025



# Reporting Summary

Nature Portfolio wishes to improve the reproducibility of the work that we publish. This form provides structure for consistency and transparency in reporting. For further information on Nature Portfolio policies, see our Editorial Policies and the Editorial Policy Checklist.

## Statistics

For all statistical analyses, confirm that the following items are present in the figure legend, table legend, main text, or Methods section.

| n/a | Confirmed | |
|---|---|---|
| ☐ | ☒ | The exact sample size ($n$) for each experimental group/condition, given as a discrete number and unit of measurement |
| ☐ | ☒ | A statement on whether measurements were taken from distinct samples or whether the same sample was measured repeatedly |
| ☐ | ☒ | The statistical test(s) used AND whether they are one- or two-sided<br>*Only common tests should be described solely by name; describe more complex techniques in the Methods section.* |
| ☒ | ☐ | A description of all covariates tested |
| ☒ | ☐ | A description of any assumptions or corrections, such as tests of normality and adjustment for multiple comparisons |
| ☐ | ☒ | A full description of the statistical parameters including central tendency (e.g. means) or other basic estimates (e.g. regression coefficient) AND variation (e.g. standard deviation) or associated estimates of uncertainty (e.g. confidence intervals) |
| ☐ | ☒ | For null hypothesis testing, the test statistic (e.g. $F$, $t$, $r$) with confidence intervals, effect sizes, degrees of freedom and $P$ value noted<br>*Give P values as exact values whenever suitable.* |
| ☒ | ☐ | For Bayesian analysis, information on the choice of priors and Markov chain Monte Carlo settings |
| ☒ | ☐ | For hierarchical and complex designs, identification of the appropriate level for tests and full reporting of outcomes |
| ☐ | ☒ | Estimates of effect sizes (e.g. Cohen's $d$, Pearson's $r$), indicating how they were calculated |

*Our web collection on statistics for biologists contains articles on many of the points above.*

## Software and code

Policy information about availability of computer code

| | |
|---|---|
| Data collection | This study did not collect data. No software was used for data collection. |
| Data analysis | Data were analyzed using Python 3.9 and Pytorch 1.13. Two-step tasks for meta-reinforcement learning were implemented in NeuroGym (version 0.0.1). Custom Code related to this study is available at https://github.com/jil095/tinyRNN. |

For manuscripts utilizing custom algorithms or software that are central to the research but not yet described in published literature, software must be made available to editors and reviewers. We strongly encourage code deposition in a community repository (e.g. GitHub). See the Nature Portfolio guidelines for submitting code & software for further information.

## Data

Policy information about availability of data

All manuscripts must include a data availability statement. This statement should provide the following information, where applicable:

- Accession codes, unique identifiers, or web links for publicly available datasets
- A description of any restrictions on data availability
- For clinical datasets or third party data, please ensure that the statement adheres to our policy

All datasets used in this study are publicly available.The monkey dataset on the reversal learning task (Bartolo et al.) is available at https://data.mendeley.com/datasets/p7ft2bvphx.The rat dataset on the two-stage task (Miller et al.) is available at https://doi.org/10.6084/m9.figshare.20449140.The three mouse datasets on the reversal learning task, two-stage task, and transition-reversal two-stage task (Akam et al.) are available at https://osf.io/8jwhm.The human dataset on the three-

armed reversal learning task (Suthaharan et al.) is available at https://github.com/psuthaharan/covid19paranoia. The human dataset on the four-armed drifting bandit task (Bahrami et al.) is available at https://osf.io/f3t2a.The human dataset on the original two-stage task (Gillan et al.) is available at https://osf.io/usdgt.

## Research involving human participants, their data, or biological material

Policy information about studies with human participants or human data. See also policy information about sex, gender (identity/presentation), and sexual orientation and race, ethnicity and racism.

| | |
|---|---|
| Reporting on sex and gender | This study did not collect data from human subjects. Public human datasets were used. |
| Reporting on race, ethnicity, or other socially relevant groupings | This study did not collect data from human subjects. Public human datasets were used. |
| Population characteristics | This study did not collect data from human subjects. Public human datasets were used. |
| Recruitment | This study did not collect data from human subjects. Public human datasets were used. |
| Ethics oversight | This study did not collect data from human subjects. Public human datasets were used. |

Note that full information on the approval of the study protocol must also be provided in the manuscript.

# Field-specific reporting

Please select the one below that is the best fit for your research. If you are not sure, read the appropriate sections before making your selection.

☒ Life sciences ☐ Behavioural & social sciences ☐ Ecological, evolutionary & environmental sciences

For a reference copy of the document with all sections, see nature.com/documents/nr-reporting-summary-flat.pdf

# Life sciences study design

All studies must disclose on these points even when the disclosure is negative.

| | |
|---|---|
| Sample size | We analyzed preexisting behavioral datasets from multiple species and tasks:<br>- For the reversal learning task, we analyzed data from two monkeys (Bartolo dataset) and ten mice (Akam dataset).<br>- For the two-stage task, we analyzed data from four rats (Miller dataset) and ten mice (Akam dataset).<br>- For the transition-reversal two-stage task, we analyzed data from seventeen mice (Akam dataset).<br>- For human participants, we analyzed data from 1,010 individuals performing the three-armed reversal learning task (Suthaharan dataset), 975 individuals performing the four-armed drifting bandit task (Bahrami dataset), and 1,961 individuals performing the original two-stage task (Gillan dataset).<br>No statistical methods were used to predetermine sample sizes. All datasets were drawn from previously published studies, and we included all available subjects (with enough trials for modeling) in each task. For animal datasets, our analyses focused primarily on individual-level behavioral effects, where sample size is less revelant compared to group-level effects. For human datasets, we analyzed the largest publicly available datasets for each task, with sample sizes exceeding those used in most prior studies. These large cohorts provide sufficient statistical power to support robust group-level inferences and model-based analyses. |
| Data exclusions | Two rats (Miller dataset) performing the two-stage task were excluded and not analyzed due to the insufficient number of trials (each animal having fewer than 2000 trials). 57 participants (5.9%) who missed more than 10% of trials were excluded from Bahrami dataset.<br>No subject was excluded from other datasets. |
| Replication | Our analysis is based on individual-level data from publicly available behavioral datasets, originally collected in independently performed and previously published studies. While we did not perform new experimental replications ourselves, our modeling conclusions were robustly replicated across multiple independent datasets (eight datasets in total), five experimental paradigms, spanning three animal species (monkeys, mice, rats) and thousands of human participants. This extensive replication across independently conducted experiments demonstrates the generalizability and reproducibility of our findings. |
| Randomization | The datasets analyzed were derived from publicly available behavioral experiments previously reported in the literature. Allocation to experimental groups was not randomized by us; instead, randomization was previously performed by the original authors. As our modeling approach focuses on computational characterization of individual cognitive strategies rather than causal inferences across experimental groups, controlling additional covariates or further randomization is not relevant. |
| Blinding | The present study involves computational modeling and analysis of existing, publicly available behavioral datasets. It does not include any direct behavioral experimentation. Therefore, blinding was not required. |

# Reporting for specific materials, systems and methods

We require information from authors about some types of materials, experimental systems and methods used in many studies. Here, indicate whether each material, system or method listed is relevant to your study. If you are not sure if a list item applies to your research, read the appropriate section before selecting a response.

## Materials & experimental systems

| n/a | Involved in the study |
|---|---|
| ☒ | ☐ Antibodies |
| ☒ | ☐ Eukaryotic cell lines |
| ☒ | ☐ Palaeontology and archaeology |
| ☒ | ☐ Animals and other organisms |
| ☒ | ☐ Clinical data |
| ☒ | ☐ Dual use research of concern |
| ☒ | ☐ Plants |

## Methods

| n/a | Involved in the study |
|---|---|
| ☒ | ☐ ChIP-seq |
| ☒ | ☐ Flow cytometry |
| ☒ | ☐ MRI-based neuroimaging |
